# CDK4/6 inhibition in luminal breast cancer

**DOI:** 10.1007/s12254-016-0268-2

**Published:** 2016-06-20

**Authors:** Simon Peter Gampenrieder, Gabriel Rinnerthaler, Richard Greil

**Affiliations:** IIIrd Medical Department with Hematology and Medical Oncology, Oncologic Center, Paracelsus Medical University Salzburg, Müllner Hauptstraße 48, 5020 Salzburg, Austria; Laboratory of Immunological and Molecular Cancer Research, Center for Clinical Cancer and Immunology Trials, Salzburg Cancer Research Institute, Salzburg, Austria; Cancer Cluster Salzburg, Salzburg, Austria

**Keywords:** CDK4/6 inhibitors, Palbociclib, Ribociclib, Abemaciclib, Endocrine resistance

## Abstract

Endocrine therapy represents the basis for the treatment of estrogen receptor-positive breast cancer, but several tumors harbor intrinsic resistance and acquired resistance to endocrine therapy is inevitable in metastatic disease. Combination strategies of endocrine therapy with targeted agents are aimed to overcome endocrine resistance. The selective CDK4/6 inhibitor palbociclib has shown promising results in metastatic luminal breast cancer when used in combination with endocrine therapy both in the first-line setting as in pretreated women. The drug showed a manageable safety profile with uncomplicated neutropenia as the most frequent side effect. Approval was already granted in the US and is also awaited during 2016 for Europe.

## Introduction

Loss of normal cell cycle regulation is one of the hallmarks of cancers [1] and alterations of the components involved in cell cycle control occur frequently in breast cancer [2]. Therefore, inhibition of cyclin dependent kinases (CDKs) is an attractive approach in tumor therapy. Unselective CDK inhibitors, however, have only limited activity in several tumor entities [3]. In contrast, selective CDK4/6 inhibitors show a very favorable toxicity profile in addition to a remarkable efficacy when combined with endocrine therapy in the treatment of metastatic luminal breast cancer [4, 5]. Here we review the mechanism of action, the rationale for the use in luminal breast cancer, and the available clinical data for CDK4/6 inhibitors.

## Mechanism of action

**Fig. 1:**
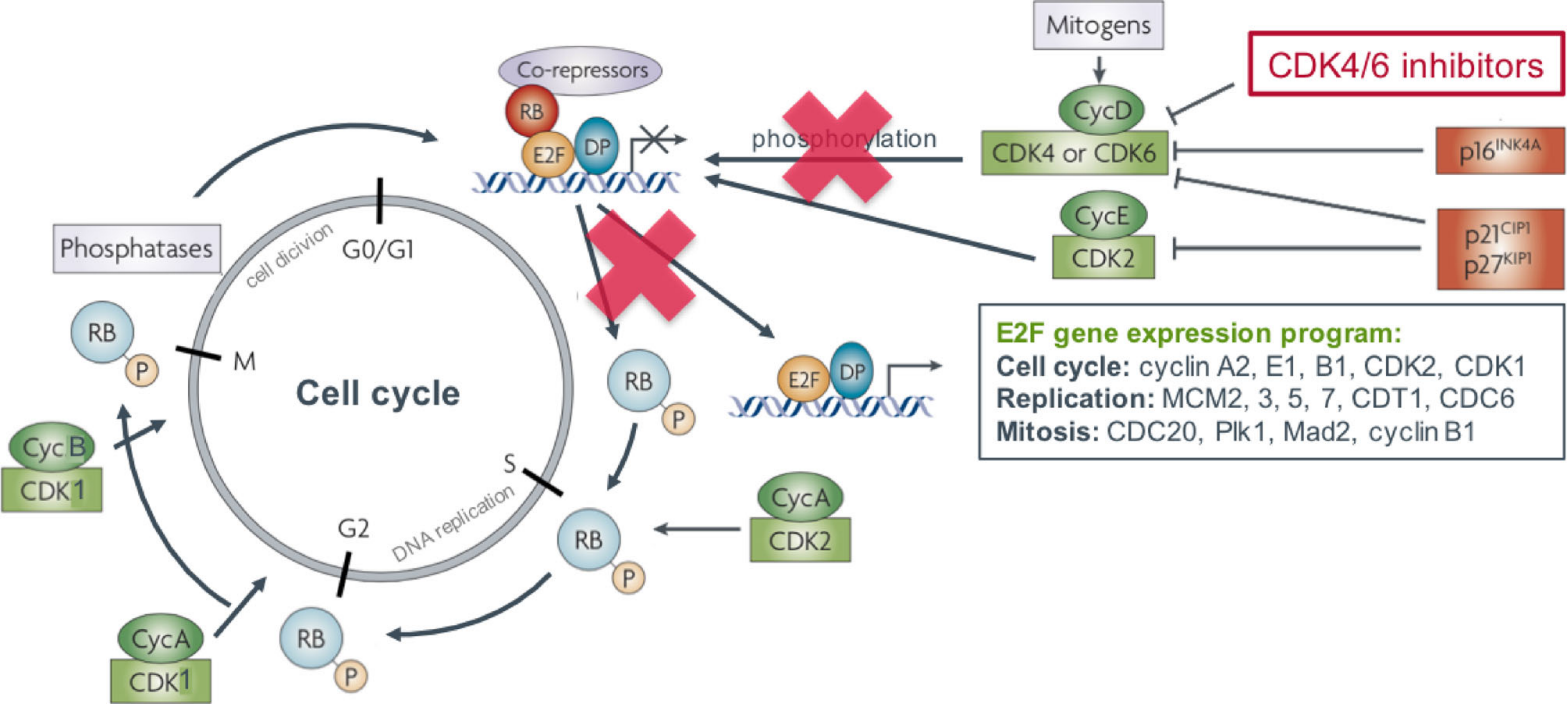
Mechanism of action (adapted from Knudsen & Knudsen. Nat Rev Cancer 2008 [6])

Retinoblastoma (RB) captures a key function in cell cycle control [6]. Under normal conditions the unphosphorylated RB is bound to members of the transcription factor family E2F, which in turn are linked to a protein called dimerization partner (DB). Mitogenic signals stimulate the expression of cyclin D, which activates the cyclin-dependent kinases 4 (CDK4) and 6 (CDK6), resulting in the phosphorylation of RB. Thereby RB disassociates from E2F allowing the transcription of E2F-dependent genes. Other cyclin-CDK complexes (CDK2/cyclin E or cyclin A, CDK1/cyclin A or cyclin B) augment this phosphorylation process. E2F induces the expression of cell cycle genes, including cyclins and CDKs, and of genes involved in replication and mitosis enabling progression from G1-phase to S‑phase and to mitosis (M-phase). At the transition from M‑ to G1-phase, RB is dephosphorylated and thereby activated again by phosphatases. Importantly, the cell cycle is negatively regulated by intrinsic CDK inhibitors. Two families of such CDK inhibitors are known: (1) INK4 (p16^Ink4a^, p15^Ink4b^, p18^Ink4c^, p19^Ink4d^) inhibiting the cyclin D dependent kinases CDK2, -4, and -6 and (2) Cip/Kip (p21^cip1^, p27^kip1^, p57^kip2^) inhibiting the cyclin E and A dependent CDK2 (Fig. 1; [6–8]).

## Why is CDK4/6 inhibition reasonable in luminal breast cancer?

**Fig. 2:**
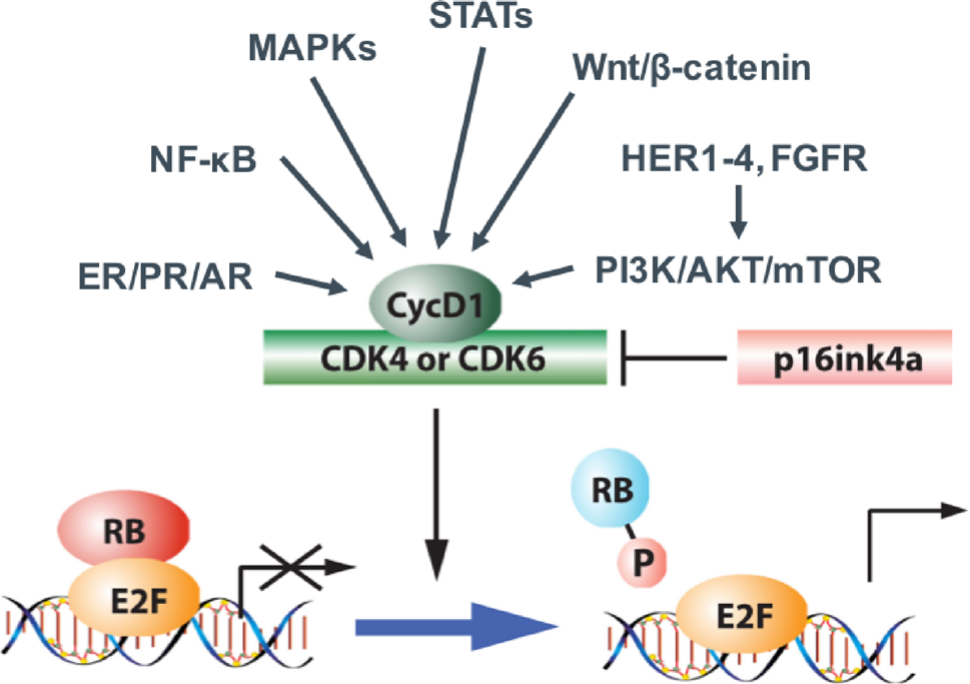
Many known drivers of breast cancer result in cyclin D regulation (adapted from Witkiewicz AK & Knudsen ES. Breast Cancer Res 2014 [8])

In breast cancer, many known drivers like estrogen receptor (ER) or HER2 lead to cyclin D accumulation. Furthermore, important signaling pathways like PI3K (phosphoinositide 3‑kinase), MAPK (mitogen-activated protein kinases), STAT (signal transducer and activator of transcription), Wnt, and NFkB (nuclear factor kappa-light-chain-enhancer of activated B cells) converge to cyclin D and cause cell cycle progression (Fig. 2; [8, 9]).

In addition, the CDK/RB pathway is frequently dysregulated in breast cancer either by cyclin D amplification or overexpression, loss of RB through deletion, mutation or gene methylation or due to p16 deletion or methylation [2, 7, 8]. The frequency of these different mechanisms of cell cycle deregulation strongly differs by breast cancer subtype: while cyclin D overexpression is frequent in luminal tumors (especially in luminal B tumors—up to 40 %) and rare in triple negative breast cancer (TNBC), RB loss is mainly restricted to nonluminal tumors [8]. Not surprisingly, such alterations of cell cycle regulators are associated with worse prognosis in ER-positive breast cancer [8] and are known mechanism of endocrine resistance [10]. Consequently, CDK4/6 inhibition seems a reasonable target for breast cancer treatment. Preclinical data show that the sensitivity to CDK4/6 inhibition is restricted to luminal and HER2 amplified cell lines, while nonluminal cell lines harbor intrinsic resistance [11]. This fact can partly be explained by the higher frequency of RB loss in nonluminal tumors [8]. Since RB is the main target of CDK4/6, cancers that have lost RB, proliferate independently of CDK4 and -6 and consecutively do not respond to CDK4/6 inhibition [12]. Furthermore, a synergistic effect of CDK4/6 inhibitors and endocrine therapy has been shown in preclinical models [11].

## Clinical data

Three CDK4/6 inhibitors are currently under clinical development: abemaciclib, ribociclib, and palbociclib (Table 1). All three compounds showed promising results in phase I trials and many phase II and phase III trials are ongoing. To date clinical data beyond phase I are only available for palbociclib.

**Table 1:** CDK4/6 inhibitors under clinical development

**Drug**	**Company**	**Phase**
Abemaciclib (LY2835219)	Lilly Oncology	III ongoing (MONARCH trials)
Ribociclib (LEE011)	Novartis	III ongoing (MONALEESA trials)
Palbociclib (PD0332991)	Pfizer	III ongoing (PALOMA trials) II and III published

## Efficacy

### Single agent phase II trial.

In a single-arm phase II trial 32 patients with metastatic breast cancer positive for RB protein were treated with palbociclib 125 mg orally on days 1– 21 of a 28-day cycle. Most patient had ER-positive/HER2-negative disease (84 %), 5 % had ER-positive/HER2-positive breast cancer and 11 % suffered from TNBC. The median number of prior cytotoxic regimens was two. Objective tumor response, the primary endpoint of the study, was achieved in 2 patients (5 %) with ER-positive breast cancer. Five further patients with ER-positive disease had stable disease for at least 6 months, resulting in a clinical benefit rate (CBR) of 19 %. Overall median progression-free survival (PFS) was 3.7 months (95 % CI 1.9–5.1). In the ER-positive subgroup, PFS was longer compared to the ER-negative subgroup (*P* = 0.03) and those who had previously progressed on endocrine therapy for advanced disease (*P* = 0.02). Several biomarker candidates like RB expression and localization, Ki-67, p16 loss, and CCND1 (gene encoding cyclin D1) amplification were investigated, but failed, however, to identify a sensitive tumor population [13].

### PALOMA-1 phase II trial.

**Fig. 3:**
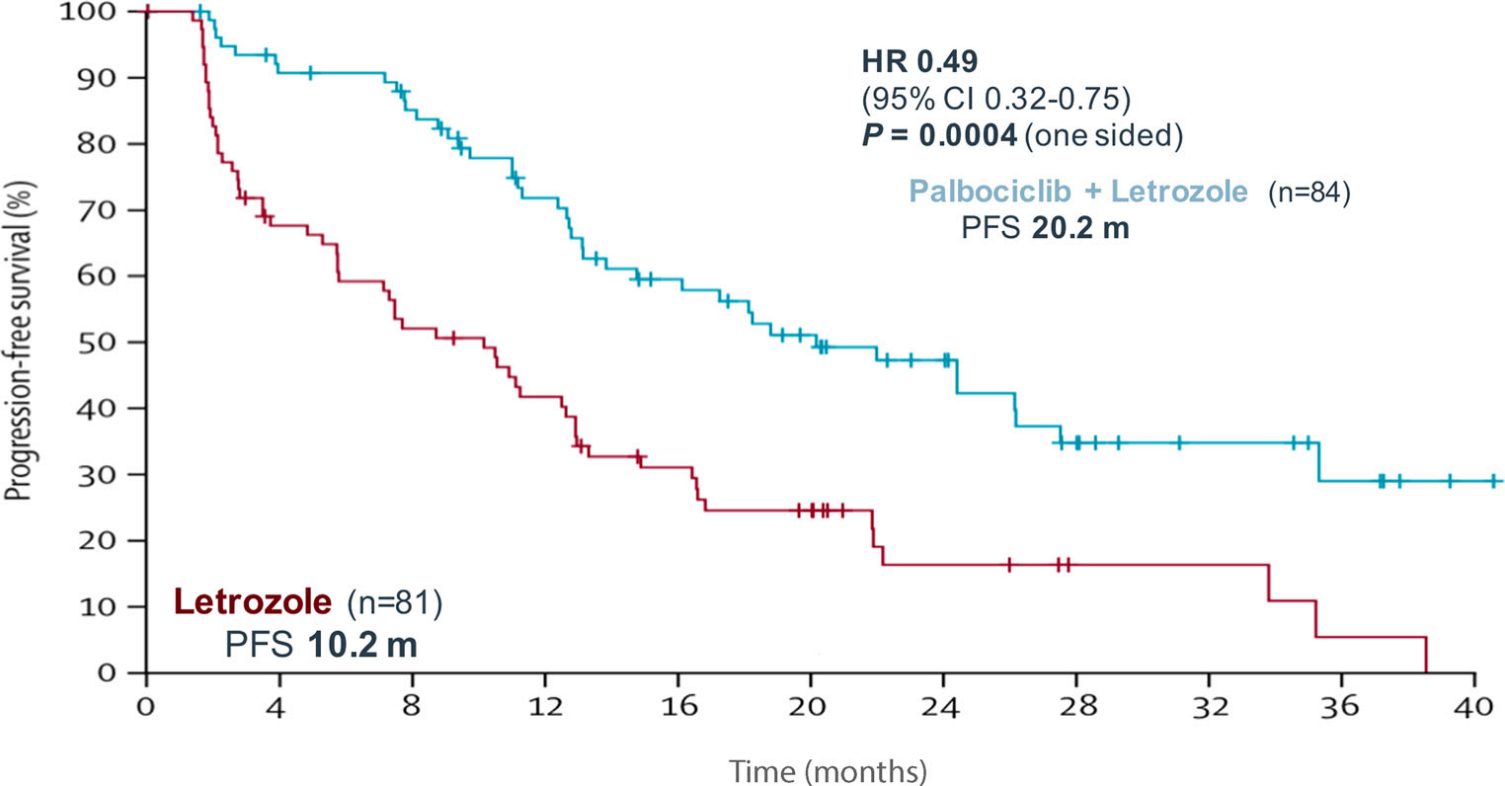
Progression-free survival (*PFS*) in the PALOMA-1 phase II trial comparing palbociclib plus letrozole with letrozole alone in the first-line setting [4]. *HR* = Hazard Ratio, *CI* = Confidence interval, *PFS* = Progression-free surivival

**Fig. 4:**
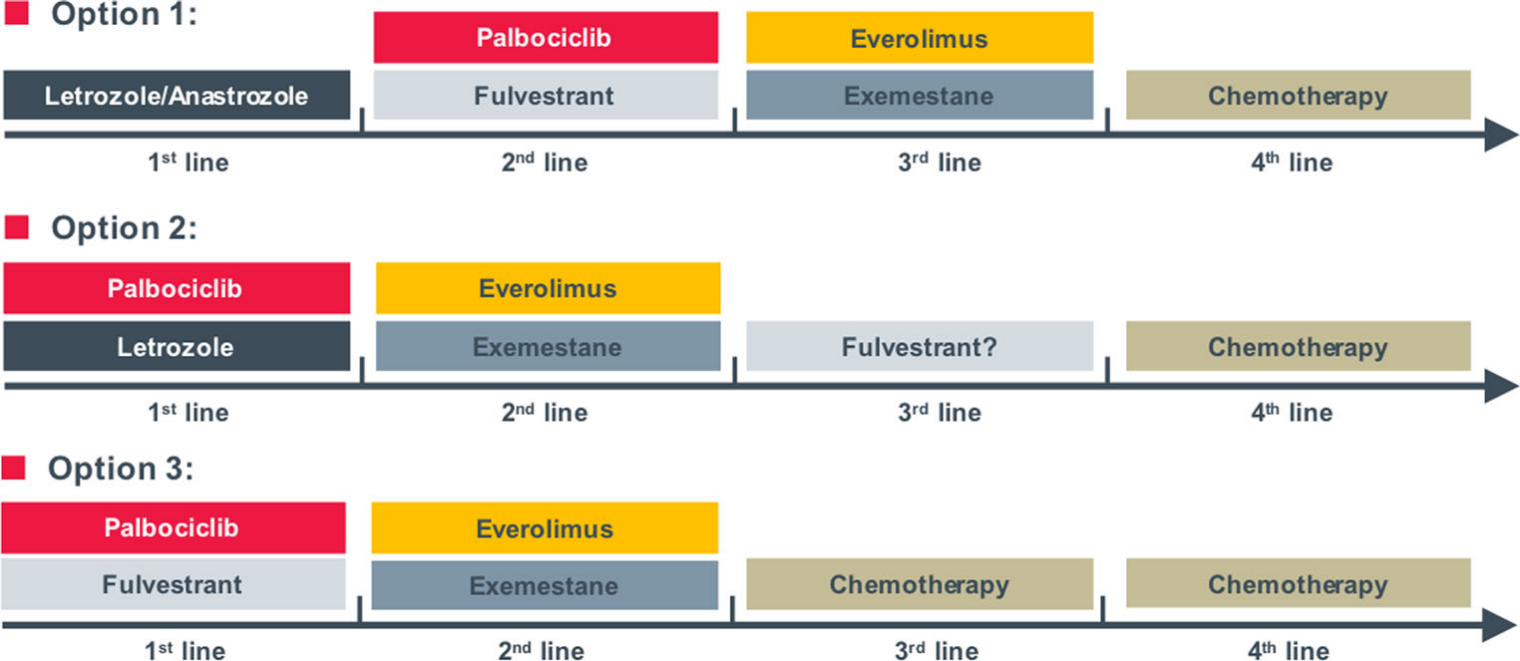
Possible treatment scenarios after approval of palbociclib

The open-label, randomized phase 2 study PALOMA-1 included 165 postmenopausal women with advanced ER-positive/HER2-negative breast cancer who had not received any systemic treatment for their advanced disease. Patients were enrolled in two separate cohorts: in cohort 1 without biomarker selection and in cohort 2 with selection for tumors with CCND1 amplification, loss of p16^Ink4a^, or both. All patients were randomly assigned 1:1 to receive letrozole 2.5 mg daily or letrozole 2.5 mg daily plus palbociclib 125 mg, given orally once daily on days 1–21 of a 28-day cycle. A third of patients had received adjuvant endocrine treatment (17 % with aromatase inhibitors). After a median follow-up of 29 months, median PFS was 10.2 months for the letrozole group and 20.2 months for the palbociclib plus letrozole group (HR 0.488, 95 % CI 0.319 to 0.748; one-sided *P* = 0.0004; Fig. 3; [4]).

The superiority of the combination treatment was consistent across all demographic subgroups and baseline prognostic factors, apart from patients with a disease-free survival (DFS) of 12 months or less from the end of adjuvant treatment. Because this subgroup consisted of 29 patients only, this has to be interpreted with caution. The number of patients with disease stabilization for at least 6 months was significantly higher in the combination arm compared to letrozole alone, resulting in a CBR of 81 % vs. 58 % (*P* = 0.0009). In contrast, the overall response rate (ORR) was not statistically different between the treatment arms (43 % vs. 33 %, *P* = 0.13). Also this trial failed to establish a biomarker for CDK4/6 inhibition. The hazard ratio (HR) for PFS was even lower in cohort 1, where patients were included irrespective of cyclin D and p16 expression within the tumor, compared to cohort 2 (HR 0.299, 95 % CI 0.156 to 0.572 and HR 0.508, 95 % CI 0.303 to 0.853, respectively) [4].

### PALOMA-3 phase III trial.

The placebo-controlled, multicenter phase III trial PALOMA-3 included 521 patients with advanced ER-positive/HER2-negative breast cancer that had relapsed or progressed during prior endocrine therapy. Patients were randomly assigned in a 2:1 ratio to receive palbociclib and fulvestrant or placebo and fulvestrant until disease progression. Interestingly this study included premenopausal women as well, who additionally received goserelin in both treatment arms. The median PFS was shorter compared to the first-line trial PALOMA-1, but the hazard ratio was in the same range (9.2 months in the experimental arm vs. 3.8 months in the control arm; HR 0.42; 95 % CI 0.32 to 0.56; *P* < 0.001). Also in PALOMA-3 all patient subgroups benefited from the addition of the CDK4/6 inhibitor to endocrine therapy. Interestingly, the smallest benefit was again in patients with a short DFS (≤24 months) with a HR of 0.84 (95 % CI 0.41–1.75). The percentage of patients achieving tumor response was low and not statistically significant different between the two treatment arms (10 % and 6 %). The CBR was 34 % in the palbociclib arm compared to 19 % in the fulvestrant alone arm (*P* < 0.001) [5].

## Safety

All three clinical trials showed a very similar toxicity profile of palbociclib [4, 5, 13]. The most common adverse events were myelosuppression with neutropenia, leukopenia, anemia, and thrombocytopenia as well as fatigue and nausea. In the PALOMA-3 phase III trial grade 3/4 neutropenia occurred in 62 % of the patients receiving palbociclib in combination with fulvestrant. Interestingly, the incidence of febrile neutropenia was very low with 0.6 % in both treatment arms. The overall frequency of infections was slightly higher in the palbociclib-fulvestrant group compared with the placebo-fulvestrant group (34 % vs. 24 %). The infections, mainly affecting the upper respiratory tract, were mostly grade 1 and 2 (Table 2; [5]). No grade 3 or grade 4 non-hematologic adverse events were reported in more than 2 % of patients receiving palbociclib, both in the PALOMA-1 and 3 trial [4, 5].

**Table 2:** Most frequent toxicities in the PALOMA-3 phase III trial (*n* = 517) [5]

	**Palbociclib + Fulvestrant (** ***n*** **= 345)**			**Placebo + Fulvestrant (** ***n*** **= 172)**		
	All in %	Grade 3 in %	Grade 4 in%	All in %	Grade 3 in %	Grade 4 in %
Neutropenia	79	53	9	4	–	<1
Leukopenia	46	25	<1	4	–	<1
Anemia	26	3	–	10	2	–
Thrombocytopenia	19	2	<1	–	–	–
Fatigue	38	2	–	27	1	–
Nausea	29	–	–	26	<1	–
Headache	21	<1	–	17	–	–
Upper respiratory infection	19	<1	–	16	–	–
Diarrhea	19	–	–	17	<1	–
Constipation	17	–	–	14	–	–
Alopecia	15	–	–	6	–	–

## Why is CDK4/6 inhibition not more toxic?

The central role of CDK4 and CDK6 in cell cycle control would suggest an unfavorable toxicity profile of CDK4/6 inhibitors. In particular, because CDK4 and CDK6 were thought to be essential for initiation of the cell cycle in response to mitogenic stimuli. A knock-out mice model, however, showed that CDK4/6-null mice embryos display normal organogenesis and most cell types proliferate normally. Nevertheless, the embryos die in late gestation due to severe anemia [14]. This experiment shows that hematopoietic cells are dependent on CDK4/6 for cell cycle entry, while other cells dispose of alternative mechanisms to initiate cell proliferation upon mitogenic stimulation. Consistently, the main toxicity of palbociclib and other CDK4/6 inhibitors is bone marrow suppression. Despite in part high-grade leukopenia and neutropenia, the incidence of febrile neutropenia seems not to be increased [4, 5]. This observation can probably be explained by the different mechanisms of myelosuppression of CDK4/6 inhibition and chemotherapy: bone marrow suppression by CDK4/6 inhibition occurs through cell cycle arrest without apoptosis which is reversible upon palbociclib withdrawal, while treatment with chemotherapeutic agents like paclitaxel or doxorubicin results in DNA damage and apoptotic cell death causing prolonged cell growth inhibition [15]. Furthermore, study protocols dictate a consequent side effect management with treatment interruptions and dose reductions in case of high grade cytopenia. The frequency of dose reductions in the PALOMA-3 trial was about 30 % [5]. An analogous careful management is required in the daily routine in order to keep the time of grade 4 neutropenia short and to prevent a higher rate of neutropenic fever as observed in the clinical trials.

## Future directions

Palbociclib is already approved by the US Food and Drug Administration (FDA) as first-line therapy in combination with letrozole in ER-positive metastatic breast cancer. Approval by the European Medicines Agency (EMA) is expected during 2016. After approval, several treatment scenarios are supposable (Fig. 4); however, trials to investigate the best treatment sequence in ER-positive metastatic breast cancer are warranted. Especially the question if endocrine monotherapy is still an option after progression to a CDK4/6 inhibitor combination remains open.

Preclinical data suggest that the main mechanism of resistance to CDK4/6 inhibitors is the upregulation of alternate cell cycle components like cyclin E1 and CDK2 [16–18]. Also increased cyclin D1 levels have been described after acquired resistance to CDK4/6 inhibitors [16, 18]. Furthermore, changes in cytoskeletal organization by Rho/Rac pathway activation were observed [18]. Interestingly, also activation of the PI3K-AKT-mTOR pathway seems to be a mechanism of resistance to palbociclib, which can be targeted with PI3K- and mTOR inhibitors, respectively [18]. Conversely, rephosphorylation of retinoblastoma was described in PI3K inhibitor resistant cell lines, making them sensitive to inhibition of CDK4/6 [19]. Thus, preclinical evidence exists for CDK4/6 inhibition before PI3K-mTOR inhibition and vice versa. Additionally, preclinical models show a synergistic effect of CDK4/6- and mTOR- or PI3K inhibitors [19, 20]. If this observation proves true on a patient level and if the combination is safe has to be demonstrated in the clinical phase I/II trials that were already initiated (ClinicalTrials.gov identifier NCT01857193, NCT01872260, NCT02088684, NCT02154776).

In parallel, two adjuvant trials with palbociclib in combination with endocrine therapy are recruiting: the PENELOPE-B trial including patients with ER-positive tumors and significant residual disease after neoadjuvant chemotherapy (NCT01864746) and the PALLAS trial including pre- and postmenopausal women with ER-positive stage II or III tumors (NCT02513394).

## Conclusion

CDK4/6 inhibitors like palbociclib enlarge the treatment armamentarium for ER-positive, HER2-negative breast cancer. Because of the favorable toxicity profile and the significant prolongation of PFS, the combination of a CDK4/6 inhibitor with endocrine therapy will most probably be the first-line therapy of choice for most patients with advanced luminal breast cancer. The results of the corresponding phase III first-line trials (PALOMA-2, MONALEESA-2, and MONARCH-3) are awaited soon and approval for palbociclib by the EMA is expected during 2016. The best endocrine partner (aromatase inhibitor [AI] vs. fulvestrant) has to be established and trials investigating treatment options after acquired resistance to CDK4/6 inhibition are required.
